# Pain Relief, Disability, and Hospital Costs After Intradiscal Ozone Treatment or Microdiscectomy for Lumbar Disc Herniation: A 24-Month Real-World Prospective Study

**DOI:** 10.3390/jcm14134534

**Published:** 2025-06-26

**Authors:** Sara Bisshopp, Renata Linertová, Miguel A. Caramés, Adam Szolna, Ignacio J. Jorge, Minerva Navarro, Brian Melchiorsen, Benjamín Rodríguez-Díaz, Jesús M. González-Martín, Bernardino Clavo

**Affiliations:** 1 Neurosurgery, Dr. Negrín University Hospital, 35019 Las Palmas, Spain; sarabisshop@gmail.com (S.B.); azszolna@gmail.com (A.S.); melchiorsenbrian@gmail.com (B.M.); 2 Fundación Canaria Instituto de Investigación Sanitaria de Canarias (FIISC), 35019 Las Palmas de Gran Canaria, Spain; benjamin.rodriguezdiaz@sescs.es (B.R.-D.); josu.estadistica@gmail.com (J.M.G.-M.); 3 Grupo BioPharm, Spain Universidad de Las Palmas de Gran Canaria, 35016 Las Palmas, Spain; 4 Evaluation Unit of the Canary Islands Health Service (SESCS), Tenerife, 38109, Spain; 5 The Spanish Network of Agencies for Health Technology Assessment and Services of the National Health System (RedETS), 28014 Madrid, Spain; 6 Network for Research on Chronicity, Primary Care, and Health Promotion (RICAPPS), 08007 Barcelona, Spain; 7 Chronic Pain Unit, Dr. Negrín University Hospital, 35019 Las Palmas, Spain; micarames@yahoo.com (M.A.C.); ijja36@gmail.com (I.J.J.); minervanavarrorivero@gmail.com (M.N.); 8 Research Unit, Dr. Negrín University Hospital, 35019 Las Palmas, Spain; 9 Instituto Universitario de Enfermedades Tropicales y Salud Pública de Canarias, Universidad de La Laguna, 38296 La Laguna, Spain; 10 CIBER de Enfermedades Infecciosas (CIBERINFEC), Instituto de Salud Carlos III, 28029 Madrid, Spain

**Keywords:** costs, discectomy or microdiscectomy, disc herniation, intradiscal ozone therapy, lumbar pain, radicular pain

## Abstract

**Background/Objectives**: Surgery is the treatment of choice for symptomatic disc herniation after unsuccessful conservative management. This prospective study compared the impact on clinical and hospital outcomes of intradiscal ozone treatment vs. surgery (microdiscectomy/discectomy) in our clinical practice. **Methods**: Intradiscal ozone treatment was offered to 70 patients with scheduled surgery because of lumbar disc herniation. Initial treatment was surgery in 38 patients and ozone infiltration in 32 patients: lumbar and sciatic pain (Visual Analog Scale), Roland-Morris Disability Questionnaire score, days of hospital admission, and direct hospital costs were recorded during 24 months of follow-up. **Results**: At 12 and 24 months, lumbar pain, sciatic pain, and Roland-Morris score decreased significantly within both groups (*p* < 0.001). At 24 months, compared to the initial surgery, the initial intradiscal ozone treatment showed similar clinical outcomes with significantly lower requirements of surgery (100% versus 47%, *p* < 0.001) and lower hospital stay [median 2.5 (2–3) versus 0.5 (0–2) days, *p* < 0.001]. Direct hospital costs were significantly lower with initial ozone treatment at 12 months (*p* = 0.040). **Conclusions**: In our real-world prospective study, after 24 months of follow-up, initial intradiscal ozone treatment avoided surgery in more than half of patients and provided similar clinical outcomes with lower hospitalization requirements. In patients with lumbar disc herniation requiring surgery (microdiscectomy/discectomy), initial intradiscal ozone treatment could offer benefits for patients and healthcare service providers (NCT00566007).

## 1. Introduction

According to the Global Burden of Disease (GBD), in 2019, low back pain (LBP) was the ninth largest disease burden [1] and the most prevalent musculoskeletal disorder [2]. It is associated with a significant impact on health-related quality of life (HRQOL), leading to disability with long-term socio-economic consequences [3]. In 2016, its estimated cost in the United States was 134.5 billion USD [4].

Lumbar disc herniation (LDH) is the primary identifiable cause of LBP, resulting in nerve root compression and radicular pain, often accompanied by sensory and motor deficits. Conservative management, such as physical therapy, nonsteroidal anti-inflammatory drugs, and activity modification, is generally recommended for a minimum of 4–12 weeks, with symptoms resolving in most patients [5,6]. Surgery, primarily by discectomy/microdiscectomy, is the treatment of choice for LDH when pain or neurological symptoms are severe at presentation or when they are refractory or progressive despite conservative management [7]. However, surgery carries inherent risks and can lead to prolonged recovery times, which has resulted in a growing interest in minimally invasive and non-surgical procedures such as chemonucleolysis, electrothermal therapy, laser decompression, radiofrequency annuloplasty, and intradiscal biacuplasty [5].

Intradiscal ozone therapy (idO3T) involves injecting a mixture of oxygen and ozone into the herniated disc, which aims to reduce inflammation, relieve pain, and promote tissue repair. Over the last two decades, several randomized controlled trials (RCTs), systematic reviews, and meta-analyses have suggested the potential of idO3T [8,9,10,11,12,13,14] and paravertebral ozone infiltrations [15,16] to avoid or delay surgery for LDH.

However, despite these promising results, there is a lack of long-term, prospective data directly comparing the clinical outcomes and healthcare costs of idO3T versus discectomy/microdiscectomy to treat LDH. Previously, in a small RCT, we reported that intradiscal + foraminal ozone infiltration could offer clinical and economic benefits [12].

Our current prospective study, in patients with scheduled surgery for LDH, aimed to evaluate the impact of idO3T versus discectomy/microdiscectomy on clinical outcomes, the percentage of surgeries finally performed, days of hospital admission, and direct hospital costs. We followed these patients for 24 months to evaluate the long-term effects of each treatment approach.

## 2. Materials and Methods

**Figure 1:**
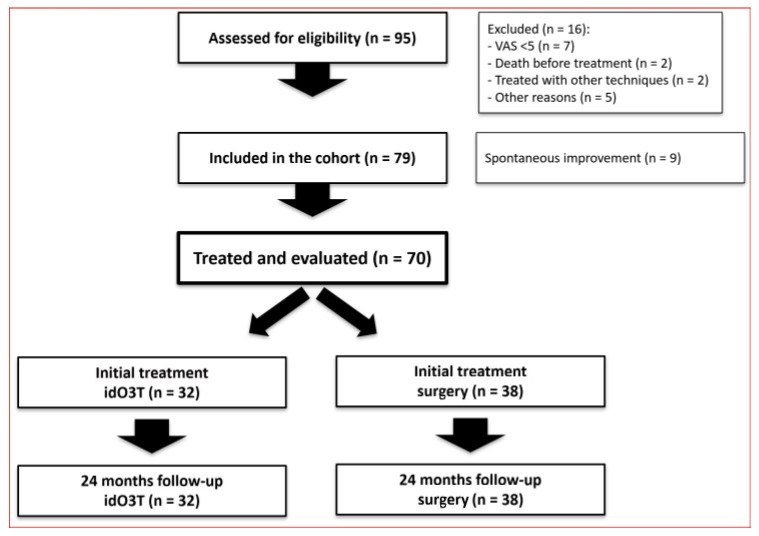
CONSORT diagram.

This prospective, non-randomized, observational cohort study, based on the results of our preliminary RCT (NCT00566007) [12], was designed to evaluate under clinical practice’s real-world conditions the effect of idO3T in the management of patients with LDH requiring discectomy/microdiscectomy, as determined by the criteria of our Department of Neurosurgery. Between March 2018 and February 2021, 95 consecutive patients from the neurosurgery waiting list for surgery were evaluated. The study was approved by the Spanish Medicines Agency (on 24 October 2016, code: BCV-OZO-2016-01) and the Research Ethics Review Committee of Las Palmas, Spain (on 27 July 2017, code: 160089/740). The study was prospectively registered (on 14 September 2017) at http://www.clinicaltrials.gov (NCT03282695) (accessed on 28 May 2025). The date of first patient enrollment was 9 March 2018. All patients provided written informed consent, and the study adhered to the Declaration of Helsinki (1975) principles. Figure 1 shows the CONSORT diagram.

### 2.1. Interventions

The main inclusion criteria for this study were (i) diagnosis of LDH associated with sciatic pain > 5 and (ii) inclusion on the waiting list for a discectomy/microdiscectomy according to traditional criteria from the Neurosurgery Department. Table 1 shows the full list of inclusion and exclusion criteria.

All patients received traditional pharmacological pain management before and after the procedures according to the criteria of their neurosurgeons and general practitioners.

idO3T infiltration was offered as a complementary treatment to the usual pharmacological drug management while patients awaited the scheduled surgery. The surgical date determined by their neurosurgeons was not modified based on whether patients chose to receive idO3T. When called for surgery, patients decided whether to proceed based on their symptoms, regardless of prior idO3T. idO3T was performed in an outpatient surgery operating room under fluoroscopy guidance (C-arm). The patient was awake under sedation in the prone position. Following aseptic measures, the appropriate entry point was in the skin to access the disc’s interior on the side where the herniated disc compressed the affected nerve root, usually outside the facet joint and with a radiological lateral inclination of about 30 degrees. We inserted a 12 cm, 20G needle, with tunnel radiological vision, into the center-lateral area of the disc. Appropriate needle placement for infiltration was controlled by antero-posterior and lateral X-ray imaging. When the needle was located at the determined target, we injected 8 mL of O3/O2 gas mixture (concentration: 28 µg/mL [µg O3/mL O2]), and at the time of needle removal, we injected an additional 4 mL of O3/O2 gas mixture (at the same concentration), steroids (4 mg dexamethasone), and anesthetic (bupivacaine at 0.25%) into the corresponding conjunction hole. Antibiotic prophylaxis was used by intravenous cefazoline (vancomycin if allergy) and streptomycin in the rinse solution. The protocol of idO3T was based on our previous RCT (registered in 2007) [12] and the study of 600 patients by Andreula et al. in 2003, which showed better results if a periganglionic injection of corticosteroid and anesthetic was added to the idO3T procedure [17].

### 2.2. Outcome Measures

The main outcome measures were as previously scheduled (NCT03282695) after 12 and 24 months of follow-up.

Clinical outcome measures were (i) lumbar and sciatic pain evaluated by the Visual Analog Scale (VAS), ranging from 0 (no pain) to 10 (the worst imaginable pain), and (ii) the Roland-Morris Disability Questionnaire (RMDQ) to evaluate the level of disability and how much patients’ daily activities are affected by their low back pain. The RMDQ includes 24 questions, with scores ranging from 0 to 24 (higher scores indicate greater disability).

The hospital outcome measures were (i) the percentage of surgeries performed, (ii) total days of hospital admission related to LDH, and iii) direct hospital costs during the follow-up. This included the cost of surgery, hospitalization, minimally invasive X-ray procedures at the Chronic Pain Unit (idO3T, foraminal, or facet blockades), medication, and medical tests. They were calculated from the patient’s medical records and the official unit costs (year of reference 2022) from the Analytical Accounting Service of Dr. Negrín University Hospital in Gran Canaria. Patients’ medical records were also used to evaluate the total required days of hospital admission related to LDH during the 12 and 24 months of follow-up. The cost of idO3T infiltration was estimated at €1274.40, based on 45 min of outpatient surgery room use at €28.32/minute. The costs for patients undergoing surgery comprised the operating room procedure fees and all related expenses from patient admission to discharge.

### 2.3. Statistical Analysis

Data was analyzed using the R Core Team 2024, version 4.4.2 software (Vienna, Austria). Statistical analysis included all inpatient days and hospital costs associated with the treatment of lumbar disc herniation (the first and the potential further treatments if required). Because most variables did not follow a normal distribution (Shapiro-Wilk test), all data were expressed as median (25th–75th percentiles) and were compared using non-parametric tests: (i) the Wilcoxon signed rank (two-tailed) for comparisons during follow-up in patients with the same initial treatment and (ii) the exact Mann–Whitney U test (two-tailed) for comparisons between treatment arms. Yates’ continuity correction was used in the comparison of proportions. *p* < 0.05 was considered statistically significant.

## 3. Results

From the 95 enrolled patients, a total of 16 patients were excluded: seven patients were scheduled for LDH surgery with VAS < 5 for sciatic pain, two patients died before receiving treatment, two patients were treated with other techniques, and five patients left the study for other reasons. Of the initial cohort of 79 patients, nine patients (11%) underwent spontaneous improvement of LDH symptoms and did not require treatment. Therefore, 70 patients were ultimately treated and evaluated according to the study protocol.

This cohort of 70 patients consisted of 42 females and 28 males, with a median age of 43 (36–51) years old and a median weight of 74 (67–83) kg. From these 70 patients, (i) 38 patients were initially treated with surgery because they rejected O3T (36 patients) or because they were called for surgery before O3T (2 patients); (ii) 32 patients accepted and were initially treated with idO3T without giving up the initially scheduled surgery.

There were no statistically significant differences between surgery and idO3T groups in age (44 (37–50) vs. 42 (36–52) years old), sex distribution (female: 63% vs. 56%), body weight (75 (67–82) vs. 73 (68–87) kg), or clinical outcome measures the day of recruitment or day of treatment. Day of recruitment: (i) lumbar-pain-VAS: 8 (7–9) vs. 7 (5.75–8); (ii) sciatic-pain-VAS: 8 (7–9) vs. 8 (7–9.25). Day of treatment: (i) lumbar-pain-VAS: 8 (7–8.75) vs. 7 (5–9); (ii) sciatic-pain-VAS: 8 (8–9) vs. 7 (5.75–9.25); and (iii) RMDQ disability: 16.5 (13–19.75) vs. 14.5 (10–19.25). However, the time from recruitment to treatment was significantly higher for surgery than for idO3T, 71 (43–131) vs. 51 (28–71) (Table 2).

**Figure 2:**
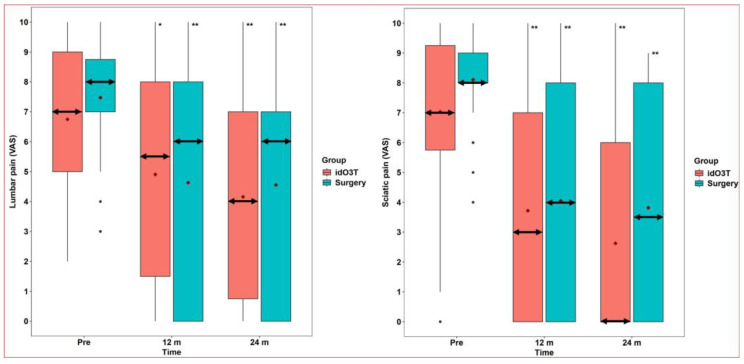
Lumbar and sciatic pains. (***Left***) *Lumbar pain.* Lumbar pain evaluated by the Visual Analog Scale (VAS) did not reveal statistically significant differences between the group initially treated by surgery and the group initially treated by intradiscal O3 infiltration (idO3T) at any time of measurement. Compared to basal values (Pre), lumbar pain was significantly lower at 12 and 24 months of follow-up in both patient groups. Box: quartiles 1 to 3. Black points: individual values. Median: double horizontal arrow. Mean: red diamond. * *p* = 0.005; ** *p* < 0.001. (***Right***) *Sciatic pain*. Sciatic pain evaluated by the Visual Analog Scale (VAS) did not reveal statistically significant differences between the group initially treated by surgery and the group initially treated by intradiscal O3 infiltration (idO3T) at any time of measurement. Compared to basal values (Pre), sciatic pain was significantly lower at 12 and 24 months of follow-up in both patient groups. Box: quartiles 1 to 3. Black points: individual values. Median: double horizontal arrow. Mean: red diamond. ** *p* < 0.001.

**Figure 3:**
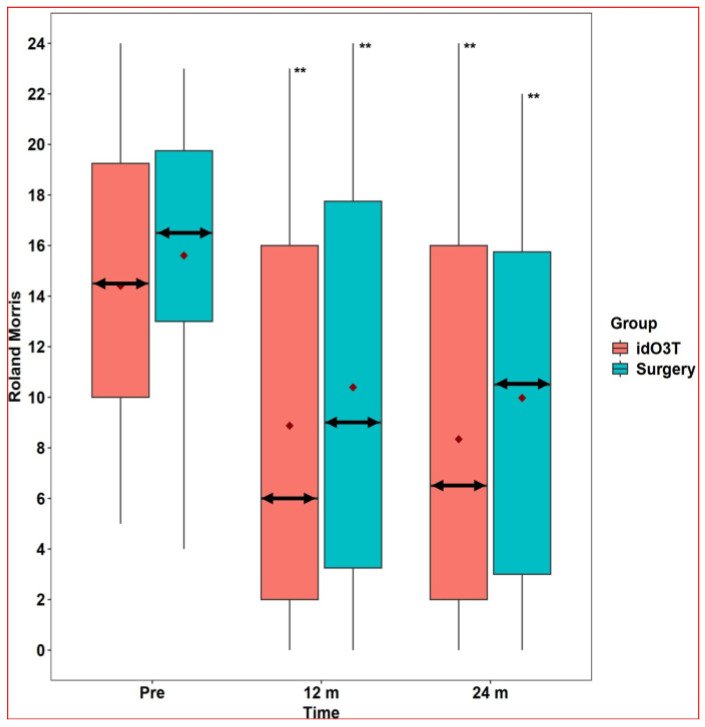
Roland-Morris Disability Questionnaire. The Roland-Morris Disability Questionnaire did not reveal statistically significant differences between the group initially treated by surgery and the group initially treated by intradiscal O3 infiltration (idO3T) at any time of measurement. Compared to basal values (Pre), the Roland-Morris Disability Questionnaire value was significantly lower at 12 and 24 months of follow-up in both patient groups. Box: quartiles 1 to 3. Black points: individual values. Median: double horizontal arrow. Mean: red diamond. ** *p* < 0.001.

At 12 months of follow-up, there were no statistically significant differences between both groups (surgery vs. idO3T) in the clinical parameters assessed: (i) VAS in lumbar pain: 6 (0–8) vs. 5.5 (1.5–8), *p* = 0.751; (ii) VAS in sciatic pain: 4 (0–8) vs. 3 (0–7), *p* = 0.651; and (iii) RMDQ disability: 9 (3.25–17.75) vs. 6 (2–16), *p* = 0.436. When values were compared with the day of treatment within each group, there was a statistically significant improvement in clinical parameters in both groups at 12 months. In the surgery group: (i) VAS in lumbar pain: 8 (7–8.75) vs. 6 (0–8), *p* < 0.001; (ii) VAS in sciatic pain: 8 (8–9) vs. 4 (0–8), *p* < 0.001; and (iii) RMDQ disability: 16.5 (13–19.75) vs. 9 (3.25–17.75), *p* < 0.001. In the O3T group: (i) VAS in lumbar pain: 7 (5–9) vs. 5.5 (1.5–8), *p* = 0.005; (ii) VAS in sciatic pain: 7 (5.75–9.25) vs. 3 (0–7), *p* < 0.001; and (iii) RMDQ disability: 14.5 (10–19.25) vs. 6 (2–16), *p* < 0.001 (Figure 2 and Figure 3).

At 24 months of follow-up, there were no statistically significant differences between both groups (surgery vs. idO3T) in clinical parameters assessed: (i) VAS in lumbar pain: 6 (0–7) vs. 4 (0.75–7), *p* = 0.53; (ii) VAS in sciatic pain: 3.5 (0–8) vs. 0 (0–6), *p* = 0.164; and (iii) RMDQ disability: 10.5 (3–15.75) vs. 6.5 (2–16), *p* = 0.376. When values were compared with the day of treatment within each group, there was a statistically significant improvement in clinical parameters in both groups at 24 months. In the surgery group: (i) VAS in lumbar pain: 8 (7–8.75) vs. 6 (0–7), *p* < 0.001; (ii) VAS in sciatic pain: 8 (8–9) vs. 3.5 (0–8), *p* < 0.001; and (iii) RMDQ disability: 16.5 (13–19.75) vs. 10.5 (3–15.75), *p* < 0.001. In the O3T group: (i) VAS in lumbar pain: 7 (5–9) vs. 4 (0.75–7), *p* < 0.001; (ii) VAS in sciatic pain: 7 (5.75–9.25) vs. 0 (0–6), *p* < 0.001; and (iii) RMDQ disability: 14.5 (10–19.25) vs. 6.5 (2–16), *p* < 0.001 (Figure 2 and Figure 3).

Comparisons between surgery and initial idO3T in lumbar pain, sciatic pain, and RMDQ at 1, 3, and 6 months after initial treatment only showed a statistically significant difference in sciatic pain at one month after initial treatment: 2.5 (0–6) vs. 6 (2.75–8), *p* = 0.023. Further details are shown in Table 2.

In the group initially treated with surgery, at 24 months, seven patients (18%) had required interventional treatment due to persistence or reappearance of symptoms: three patients (8%) had undergone a second surgery, and four patients (11%) required facet blocks in the Pain Unit as a second treatment.

In the group treated with initial idO3T, at 24 months, (i) 15 patients (47%) underwent surgery, 2 at the first month, 9 (28%) at 75 days, 10 (31%) at 6 months, and 12 (38%) at 12 months; (ii) 17 patients (53%) did not require the initially scheduled surgery, *p* < 0.001 (95% confidence interval: 33–73%).

**Figure 4:**
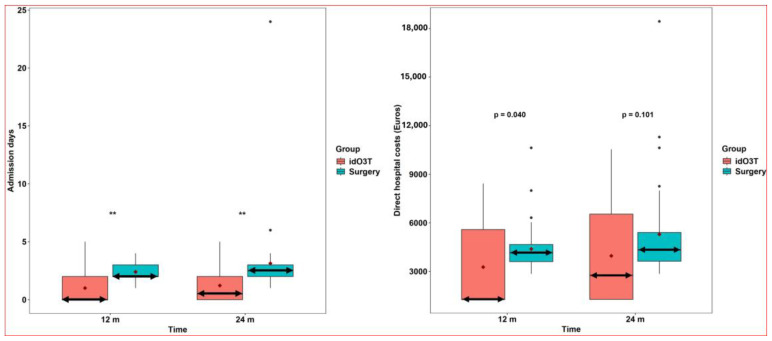
Admission days and hospital costs. (***Left***) *Days of hospital admission.* Days of hospital admission considering all hospital treatments required for the management of LDH were significantly higher in the group of patients initially treated by surgery than in the group initially treated by intradiscal O3 infiltration (idO3T): 2 days (2–3) versus 0 days (0–2) at 12 months of follow-up, and 2.5 (2–3) versus 0.5 (0–2) at 24 months of follow-up. Box: quartiles 1 to 3. Black points: individual values. Median: double horizontal arrow. Mean: red diamond. ** *p* < 0.001. (***Right***) *Direct hospital costs.* Direct hospital costs considering all hospital treatment required for the management of LDH were higher in the patient group initially treated by surgery than in the group initially treated by intradiscal O3 infiltration (idO3T): 4156.13 (3605.26–4662.95) € versus 1274.40 (1274.40–5586.78) € at 12 months of follow-up (*p* = 0.04), and 4330.45 (3631.5–5404) € versus 2757.98 (1274.40–6540.66) € at 24 months of follow-up (*p* = 0.101, not statistically significant). Box: quartiles 1 to 3. Black points: individual values. Median: double horizontal arrow. Mean: red diamond.

Days of hospital admission considering all hospital treatments required for the management of LDH were (i) at 12 months of follow-up, 2 (2–3) days in the surgery group and 0 (0–2) days in the idO3T group, *p* < 0.001; (ii) at 24 months of follow-up, 2.5 (2–3) days in the surgery group and 0.5 (0–2) days in the idO3T group, *p* < 0.001 (Figure 4).

Direct hospital costs considering all hospital treatment required for the management of LDH were (i) at 12 months of follow-up, €4156.13 (3605.26–4662.95) in the surgery group versus €1274.40 (1274.40–5586.78) in the idO3T group, *p* = 0.040; and (ii) at 24 months of follow-up, €4330.45 (3631.5–5404) in the surgery group versus €2757.98 (1274.40–6540.66) in the idO3T group (*p* = 0.101) (Figure 4).

Our study recorded no major complications associated with idO3T. The most relevant adverse event occurred in one patient of the surgery group who underwent a cerebrospinal fluid leak that required hospitalization for 2 weeks.

## 4. Discussion

This prospective study, conducted under real-world clinical practice conditions, revealed that, after 24 months of follow-up, patients with scheduled surgery for LDH initially treated with idO3T underwent similar improvements in pain and disability compared to those initially treated by discectomy/microdiscectomy. Notably, over half of the patients initially treated with idO3T avoided surgery and required significantly fewer hospitalization days without incurring higher costs.

The demonstration of similar clinical efficacy between idO3T and surgery in the mid-to-long term in our study aligns with previous findings in the few articles directly comparing idO3T versus surgery. Paradiso et al. described equivalent results between idO3T (*n* = 150) and microdiscectomy (*n* = 150), selected retrospectively from two series with 2230 patients [18]. Kelekis et al., in an RCT with 49 patients, described at 6 months that leg pain improvement after idO3T was non-inferior to microdiscectomy [13]. Finally, our own previous RCT also showed comparable clinical outcomes at 5 years [14]. Clinically, these findings are consistent with previous RCTs, systematic reviews, and meta-analyses [8,9,10,11,12,13,14].

Microdiscectomy is the gold standard for LDH when symptoms are refractory to conservative treatment. It involves the direct physical removal of the herniated disc fragment that is compressing the nerve root. Minimally invasive, non-surgical procedures for symptomatic LDH aim for pain alleviation, avoiding surgery with reduced recovery periods. Techniques such as chemonucleolysis (enzymatic dissolution of the nucleus), intradiscal electrothermal therapy (IDET), percutaneous laser disc decompression (PLDD), radiofrequency annuloplasty, and intradiscal biacuplasty share a common objective. They aim to alleviate radicular pain by reducing intradiscal pressure, ablating nociceptive nerve fibers within the annulus fibrosus, or stabilizing the disc structure through thermal coagulation. Their efficacy is variable and highly dependent on precise patient selection, generally with contained disc herniations. An RCT comparing chemonucleolysis with conventional surgery showed better outcomes at 6 weeks and 3 months in the surgery group, but the differences were not significant at 1 year. In our study, only sciatic pain at 1 month (no other clinical outcome measures) was significantly better in the surgery group, without differences at three or more months [19]. A more recent RCT comparing chemonucleolysis PLDD with conventional microdiscectomy also showed faster recovery in the surgery group but similar outcomes at 1 and 2 years. At the 2-year follow-up, surgery was avoided in nearly half of the patients (48%) in the PLDD group, similar to those described in our study [20]. Regardless, further RCTs are required to fully establish the real value of minimally invasive procedures.

A key outcome of our study is the 53% surgery avoidance rate in the idO3T group at 2 years. While the rate at 6 months was similar in our study (69%) and in the RCT by Kelekis et al. (71%) [13], the rate at two years is lower than reported in some previous studies, including our previous RCT (80% for idO3T and 40% for placebo intradiscal infiltration at 5 years) [12] and the retrospective study by Buric et al. (>80% at 5–10 years) [21]. Several factors may explain the discrepancy at two years. First, our current cohort consisted exclusively of patients already on the surgical waiting list, potentially representing more severe or refractory cases than in other series. Second, our protocol applied a single idO3T infiltration, whereas other studies allow or include multiple sessions or additional paravertebral infiltrations, which might improve the response rate. Third, a considerable proportion of patients in our idO3T group underwent surgery relatively early (28% before 2.5 months, 38% by 12 months), possibly before the full effects of idO3T could manifest. This is supported by our data showing slower improvement in sciatic pain in the idO3T group at 1 month post-treatment. Finally, the impact of the COVID-19 pandemic on patient and physician decisions regarding surgery cannot be dismissed. Additionally, related to the last two factors, a delayed improvement after idO3T has been reported in patients who had previously suffered COVID-19 [22]. Despite this more conservative surgery avoidance rate, it is important to emphasize that more than half of the patients avoided major surgical intervention, and this represents a considerable benefit.

From the perspective of hospital healthcare resource utilization, our findings are relevant. The significant reduction in cumulative hospital admission days at 24 months in the idO3T group vs. the surgery group (0.5 vs. 2.5 days) is consistent with the minimally invasive and outpatient nature of the procedure, as also described by Kelekis et al. at six months (0 vs. 2 days) [13] and our previous RCT at 5 years (0 vs. 3 days) [12]. Regarding costs, although the difference in direct hospital costs was not statistically significant at 24 months (*p* = 0.101), it was significant at 12 months, and the median cost was consistently lower in the idO3T group, as also described in our RCT14 and a large retrospective study (2589 patients) from Cuba [23]. However, a comprehensive cost-utility analysis, incorporating costs of productivity loss or informal care and utilities (Quality-Adjusted Life Years—QALYs), is required to definitively determine cost-effectiveness from a societal perspective.

In light of the preceding considerations, idO3T presents potential advantages for (i) older patients; (ii) patients with multiple comorbidities, multidrug treatment regimens, high surgical morbidity, or absolute surgical contraindications; (iii) patients expressing a preference for non-surgical management or the avoidance of hospitalization; and (iv) healthcare service providers.

The therapeutic mechanisms of idO3T are thought to involve the following: (i) Reducing intradiscal pressure, with a reduction in disc volume and nerve root compression, by the breakdown of glycosaminoglycans, with further dehydration of the nucleus pulposus [24]. (ii) Decreasing inflammation through a local decrease in oxidative stress (by overexpression of Nrf2 and subsequent increase in antioxidants) and cytokine activity (by decrease in NF-κB and proinflammatory cytokines) [24,25]. (iii) Stimulating macrophage activity and enhancing phagocytosis and resorption of the herniated disc material [25,26]. These mechanisms may explain the progressive improvement observed in our patients and the subsequent reduction in the herniated disc six months after idO3T that has been well documented by computed tomography (CT) [27] and magnetic resonance imaging (MRI) [14,21,28].

Regarding safety, when properly implemented, complications of idO3T are rare (0.1%) and minor in both general ozone therapy and disc herniation treatment, and most of the reported severe adverse events were associated with inappropriate technical procedures or malpractice rather than the ozone itself, or they did not have a causal relationship between ozone administration and the adverse event [9,21,29,30]. Hidalgo-Tallón et al. listed up to 20 reports of severe adverse events using different approaches to ozone administration [30]. The systematic review and meta-analysis of Magalhaes et al. in 2012 found six reports of side effects related to ozone administration for disc herniation [9]. In 2016, Vanni et al. briefly described eight reports [31]. The isolated serious adverse events reported included bilateral blindness, headache, neurological complications, cerebral stroke, gas embolism, and infection (discitis or even septicemia and death), although some of them were because of paravertebral infiltration, not intradiscal infiltration. Additionally, Vanny et al. reported that during microsurgery for LDH, many hard adhesions between the soft tissues and bony structures were found in patients previously treated with “intraforaminal” ozone therapy. However, those patients had received “16 intraforaminal” applications of ozone therapy in 8 weeks, which is a very different approach than one administration of intradiscal ozone infiltration [31]. This contrasts with the known complication rates of lumbar surgery (13% to 17%) [32,33], which can include dural tearing, direct nerve root injury, wound complications, local fibrosis, new or worsening neurological deficit, deep nerve thrombosis, risks of anesthesia, and the need for revision surgery (reported between 8% and 16% at 2 and 7 years of follow-up) [34].

Our study has several strengths, including its prospective design and real-world setting. However, it also has limitations. (i) It is not an RCT, and the sample size is relatively small; therefore, potential selection bias between groups cannot be excluded. Our approach was specifically chosen to provide insights into the applicability of idO3T in everyday hospital settings, especially following our preliminary RCT. Nevertheless, more RCTs with larger sample sizes are clearly required for further confirmation. (ii) The current analysis focused on direct hospital costs and did not include a comprehensive assessment of indirect costs such as medication, primary care visits, informal care, transportation, and lost productivity. (iii) Finally, the COVID-19 pandemic may have had an impact on our findings.

## 5. Conclusions

In this prospective study of surgical candidates for LDH, offering idO3T as an initial treatment resulted in comparable clinical outcomes in terms of pain and function at 24 months compared to discectomy/microdiscectomy. Importantly, it allowed more than half of the patients to avoid their scheduled surgery, with a significant reduction in hospital admission days. These findings suggest that idO3T is a viable, less invasive therapeutic alternative to surgery for selected patients, which offers potential benefits for both patients and healthcare providers in the real world. Further research is needed to confirm these findings and to establish the optimal role of idO3T in LDH management.

## Figures and Tables

**Table 1:** Inclusion and exclusion criteria.

Inclusion criteria − Patients between 18 and 75 years old diagnosed with non-calcified LDH that presents as a non-migrated protrusion and/or extrusion. − Evaluated and diagnosed by the Neurosurgery Department, having been chosen as an appropriate candidate for surgery consisting of a discectomy or microdiscectomy after meeting the following two criteria: (a) sciatic pain, with a Visual Analog Scale (VAS) intensity ≥ 5, despite 6 weeks of conservative management, whether lumbar pain exists or not, and (b) radiating pain that matches the MRI image showing one or more herniated discs. − Included in the neurosurgery waiting list for a discectomy or microdiscectomy. − Patients who have signed and dated the study’s specific informed consent.
Exclusion criteria − Patients who do not meet all the inclusion criteria. − Calcified and/or migrated herniated disc and/or with a severe neurological deficit. − LDH with indication of laminectomy and/or arthrodesis. − Relevant clinical paresis that does not improve despite 6 weeks of full conservative management. − Simultaneous symptomatic cervical or dorsal herniated discs. − Previous lumbar spine surgery. − Concomitant spine conditions that may be causing symptoms or have an indication for surgery (such as fractures or tumors). − Known allergy to ozone. − Those who are unable or do not wish to fulfill the study’s protocol or the study scales.

It was considered ‘discal protrusion’, an intervertebral disc herniation where the outermost layers of the annulus fibrosus remain intact.

**Table 2:** Details of clinical and hospital data before and after treatment.

	Initial Surgery		Initial idO3T ^a^		
	Median	Q1–Q3 ^b^	Median	Q1–Q3	*p*
					
Total patients (N)	38		32		
Female	24		18		
Male	14		14		
Age (years)	43.5	37–50	42	36–52	0.773
Weight (kg)	75	67–82	73	68–87	0.701

Day of recruitment					
Lumbar-pain-VAS	8	7–9	7	5.75–8	0.143
Sciatic-pain-VAS	8	7–9	8	7–9.25	0.213
Day of 1st treatment					
Lumbar-pain-VAS	8	7–8.75	7	5–9	0.172
Sciatic-pain-VAS	8	8–9	7	5.75–9.25	0.242
RMDQ	16.5	13–19.75	14.5	10–19.25	0.402
Days from recruitment to treatment	71	43–131	51	28–71	0.019 *

Lumbar-pain-VAS after 1st treatment					
1 Month	5	2.25–6	6	3.75–7	0.222
3 Months	5	1.25–7	5	2–7.25	0.845
6 Months	6	1–8	6	2.5–8	0.780
12 Months	6	0–8	5.5	1.5–8	0.751
24 Months	6	0–7	4	0.75–7	0.53

Sciatic-pain-VAS after 1st treatment					
1 Month	2.5	0–6	6	2.75–8	0.023 *
3 Months	2.5	0–6.75	4	1–7	0.395
6 Months	5	0–7	4	0.75–6.25	0.967
12 Months	4	0–8	3	0–7	0.651
24 Months	3.5	0–8	0	0–6	0.164

RMDQ after 1st treatment					
1 Month	8.5	6–16.75	12	4–16	0.768
3 Months	7.5	4–12.25	7	3–14.5	0.915
6 Months	9	3.25–17	6	3–12.5	0.527
12 Months	9	3.25–17.75	6	2–16	0.436
24 Months	10.5	3–15.75	6.5	2–16	0.376

Hospital admission days					
12 Months	2	2–3	0	0–2	<0.001 **
24 Months	2.5	2–3	0.5	0–2	<0.001 **

Direct hospital costs (euros)					
12 Months	4156	3605–4663	1274	1274–5587	0.040 *
24 Months	4330	3632–5404	2758	1274–6541	0.101

Surgeries at 24 months (N (%))	38 (100%)		15 (47%)		<0.001 **

^a^ idO3T: intradiscal ozone treatment; RMDQ: Roland-Morris Disability Questionnaire; VAS: Visual Analog Scale; ^b^ Q1–Q3: quartiles 1 and 3; * *p* < 0.05; ** *p* < 0.001.

## Data Availability

The raw data supporting the conclusions of this article will be made available by the authors on request.

## Abbreviations

The following abbreviations are used in this manuscript:

CT	computed tomography
GBD	global burden of disease
HRQOL	health-related quality of life
idO3T	intradiscal ozone therapy
LBP	low back pain
LDH	lumbar disc herniation
MRI	magnetic resonance imaging
QALYs	quality-adjusted life years
RCT	randomized controlled trial
